# Selecting top candidates for medical school selection interviews- a non-compensatory approach

**DOI:** 10.1186/s12909-020-02031-6

**Published:** 2020-04-15

**Authors:** Boaz Shulruf, Anthony O’Sullivan, Gary Velan

**Affiliations:** Office of Medical Education, the University of New South Wales, UNSW, Sydney, NSW 2052 Australia

**Keywords:** Admissions, Selection interview, Applicants

## Abstract

**Background:**

Medical schools apply a range of selection methods to ensure that admitted students succeed in the program. In Australia, selection tools typically include measures of academic achievement (e.g. the Australian Tertiary Admission Rank – ATAR) and aptitude tests (e.g. the Undergraduate Medicine and Health Sciences Admissions Test – UMAT). These are most commonly used to determine which applicants are invited for additional selection processes, such as interviews. However, no previous study has examined the efficacy of the first part of the selection process. In particular, are compensatory or non-compensatory approaches more effective in evaluating the outcomes of cognitive and aptitude tests, and do they affect the demographics of students selected for interview?

**Methods:**

This study utilised data from consecutive cohorts of mainstream domestic students who applied to enter the UNSW Medicine program between 2013 to 2018. A compensatory ranked selection model was compared with a non-compensatory ranked model. Initially, ATAR marks and UMAT scores for each applicant were ranked within each cohort. In the compensatory model, the mean of the ATAR and UMAT ranks were used to determine the outcome. In the non-compensatory model, the lowest rank of ATAR and UMAT determined the outcome for each applicant. The impact of each model on the gender and socioeconomic status of applicants selected to interview was evaluated across all cohorts.

**Results:**

The non-compensatory ranked selection model resulted in substantially higher ATAR and UMAT thresholds for invitation to interview, with no significant effect on the socioeconomic status of the selected applicants.

**Conclusions:**

These results are important, demonstrating that it is possible to raise the academic threshold for selection to medicine without having any negative impact on applicants from low socioeconomic backgrounds. Overall, the evidence gathered in this study suggests that a non-compensatory model is preferable for selecting applicants for medical student selection interview.

## Background

Securing a place in a medical program is one of the most competitive challenges faced by young people pursuing higher education [1]. For example at the University of New South Wales, Sydney, Australia (UNSW) only about one in ten applicants succeed in securing a place in the Medicine program. Medical schools apply a range of selection methods to ensure that admitted students succeed in the program, complete the their studies in a timely manner and become competent junior doctors [2]. The most common selection tools used by Australian undergraduate medical schools is the Australian Tertiary Admission Rank (ATAR) or equivalent, which is a score between 0.00 and 99.95 based on high school matriculation examinations, indicating a student’s position in their cohort [3]. The second selection tool, which had been used in Australia for over a decade until 2018, is the Undergraduate Medicine and Health Sciences Admission Test (UMAT) [4, 5]. The UMAT was devised by the Australian Council for Educational Research, and consists of three sections: logical reasoning and problem solving; understanding people; and non-verbal reasoning. Most undergraduate medical programs in Australia use ATAR and UMAT outcomes to select a smaller group of applicants for additional selection processes, which may include interviews, psychological tests, or both [2, 6–9]. Most of the literature focusing on the efficacy of selection to medical programs focuses on the final stage of the selection process; that is selection from the pool of applicants who were successful in being offered an interview [2, 9–11]. A few medical schools do not require an interview and use only academic and cognitive test achievements to select medical students [2, 10]. Although earlier works described different method used for the first part of the medical program selection process (i.e. selection for an interview) [12], we were not able to identify any comparable study to ours.

This gap in the literature raises concern, since it is well known that different selection tools yield different results. It is therefore possible that applicants who failed securing an offer for an interview might have been successful medical students and later on successful doctors [9, 13, 14], while places are offered to possibly less suitable applicants [2]. Moreover, the methods for selecting applicants for interviews varies across medical schools, yet no evidence has been presented regarding the efficacy of any of these methods.

### Compensatory and non-compensatory selection methods

Beyond the Structural and procedural differences across medical schools, there is an important conceptual issue that needs to be considered in the selection process: Should a compensatory or non-compensatory selection model be used? The difference between the two approaches is significant, if not critical [15]. A compensatory approach allows high scores on one selection tool to compensate for low scores on other tools. Such an approach may be based on two separate assumptions. Either that all selection tools measure approximately the same set of attributes or academic performance, and therefore the reason for using multiple measures is to increase the reliability and validity of the assessments for selection [16]; or that there is a range of combined attributes or academic performances that differ from each other, though each may indicate sufficient competence for being a successful student [17, 18].

A non-compensatory approach is based on the assumption that each selection test measures a discrete set of attributes or academic performance, each of which is important. Thus, to be a successful student, one needs to score sufficiently high on all selection tests [2]. It is expected that each approach (compensatory or non-compensatory) would select a different pool of applicants for selection interviews. The unanswered question is: Which approach is preferable, and in what circumstances? The evidence from a range of literature concerning selection to medicine suggest that the higher the score on the academic and aptitudes selection tools, the better student outcomes are observed [4, 19]. Moreover, it was demonstrated that thresholds or cut-scores for UMAT and ATAR could be useful for selection into medicine in some Australian/New Zealand programs, but not in others [2].

Apart from performance at medical school, there is also a concern for social accountability. Previous studies demonstrated that higher socioeconomic status (SES) is strongly associated with better medicine selection test scores [20, 21]. It is clear that a complex set of considerations needs to be addressed in relation to medical student selection. Succinctly, the challenge is how to improve the academic and other qualities of medical students and at the same time selecting a cohort who appropriately represents a spectrum of the community, particularly across the spectrum of SES. There is a widespread consensus that under-represented and socially disadvantaged populations should be encouraged to participate and succeed in the medical profession. Any improvement of the quality of medical student selection must also take this into consideration. Thus, it is important that the applicants selected for interview on the one hand would be those with the highest scores on all selection tools and on the other hand applicants from under-represented population would not be disadvantaged by the selection process.

The objective of this study was to test the efficacy and feasibility of using a non-compensatory approach for interview selection in comparison to the traditional compensatory approach used in a large Australian medical school.

## Methods

### Data

This study used administrative data for applicants who applied to enter the UNSW medicine program in the years 2013 to 2018. Ethics approval for this study was granted by the Human Research Ethics Committee of the University of New South Wales (ref: HC15421; see details in the ‘Ethics approval and consent to participate’ section at the end of the manuscript). The data included applicants’ UMAT scores, ATAR marks, gender and post code. The data used for this study only included those who applied to the mainstream domestic pathway. Applicant data for other pathways (international, rural and Indigenous) were excluded, since each applies different selection criteria. Socioeconomic status (SES) data were received from the Australian Bureau of Statistics (2033.0.55.001 Socio-Economic Indexes for Australia (SEIFA) released 27 March 2018). It is noted that postcode may not be very accurate for indicating SES at the individual level, but for a population study such as this, the data are sufficiently accurate [22]. The SEIFA data is based on the Australian census and considers income, occupation and education level within households. For more technical detail, please see Technical Paper Socio-Economic Indexes for Areas (SEIFA) 2011 [23].

The normal selection for interview process for the mainstream domestic pathway applies the following rules:

1. Every domestic applicant whose ATAR is ≥96.0 and average UMAT score is ≥50 is eligible to apply to medicine;
2. ATAR UMAT scores are transformed to standardised scores and the applicants are ranked by the average of the standardised UMAT and ATAR scores.

This is a compensatory selection model in which a high score on one tool may compensate for a low score on another. Each year about 400 domestic applicants are invited for an interview.

### Selection models

In this study we compared two models, a compensatory ranked model to a non-compensatory ranked model.

*The compensatory rank model* (CM) applies the following process: (a) each applicant was ranked twice, once by the UMAT scores (RU) and once by the ATAR marks (RA) (1 = top rank); (b) the mean of the two ranks comprises each applicant’s combined rank. For example, in Table 1, Applicant 5 has RU = 1 and RA = 13, thus their mean rank = (1 + 13)/2 = 7.The mean ranks are then re-ranked to create a final mean rank (MR - Table 1, column I, top rank = 1). The top 400 applicants (based on MR) are identified as invitees for interview. This is a better alternative to the standardised method used by UNSW, since the ATAR marks are actually ranks and they are not normally distributed. Mathematically, however, the *order* of the ATAR standardised scores and the ranks (RA) must be identical. Thus, the compensatory rank model (MR) is deemed representing the actual selection for interview process applied at UNSW.

**Table 1:** Demonstration of the ranking algorithm (1 = top rank)

A	B	C	D	E	F	G	H	I
Applicant	UAMT	ATAR	RU	RA	Lowest rank (1 is top rank)	**LR**	Mean rank	**MR**
11	70	96.00	4.5	5	5	**1**	4.75	**2**
2	80	94.35	2	7	7	**2**	4.5	**1**
6	68	94.35	6	7	7	**3**	6.5	**6**
10	67	94.35	7	7	7	**4**	7	**7.5**
9	66	97.00	8	3.5	8	**5**	5.75	**3.5**
13	60	98.00	10	1.5	10	**6**	5.75	**3.5**
4	59	98.00	11	1.5	11	**7**	6.25	**5**
7	70	92.00	4.5	11	11	**8**	7.75	**9**
12	58	93.00	12	9.5	12	**9**	10.75	**12**
5	85	91.15	1	13	13	**10**	7	**7.5**
14	71	91.15	3	13	13	**11**	8	**10**
3	63	91.15	9	13	13	**12**	11	**13.5**
8	54	97.00	13.5	3.5	13.5	**13**	8.5	**11**
1	54	93.00	13.5	8.5	13.5	**14**	11	**13.5**

A Applicant ID

B UMAT score

C ATAR mark

D UMAT Rank (1 is top rank)

E ATAR Rank (1 is top rank)

F Lowest Rank among UMAT & ATAR (1 is top rank)

G LR = Final Low Rank (1 is top rank)

H Mean Rank = (d + f)/2

I MR = Final Mean Rank (1 is top rank)

*The non-compensatory mode* (NCM) applies the following algorithm: Each applicant was ranked twice - once by UMAT scores (RU) and once by ATAR marks (RA) (1 = top rank). Then, the applicants were ranked again by the ***lowest*** rank of RU and RA. For example, in Applicant 5 has RU = 1 and RA = 13, thus their lowest rank (LR) is 13. If two or more applicants received the same lowest rank, then they were ordered by their highest rank (Table 1). For example, applicant 14 has also RA = 13 but RU = 3, thus is ranked lower than applicant 5 despite both having the same lowest rank. Table 1is self-explanatory and presents a number of scenarios. Similar to the MR model also for the LR model the top 400 applicants (based on LR) are identified as invitees for interview. In Table 1 LR (column G) shows the NCM rank and MR (column I) shows the CM rank.

The demographic data used was reported by each applicant. Their home postcode was used to generate a proxy for their Socioeconomic Status (SES) decile (1 = lowest; 10 = highest). SES was estimated based on aggregated SES within each postcode area [24]. It is acknowledged that postcode is not the optimal measure for SES at the individual level [25]. However, this was the best available data for this study and is acceptable for estimating SES for population samples at a size comparable to this study [26, 27].

### Statistical analysis

The models were applied to each cohort separately; and then the top 400 applicants by model and year were marked as invitees. Comparison was made between models by year and overall for the entire sample, which covers six cohorts of applicants.

Descriptive statistics were used to analyse the applicants by cohorts and by selection characteristics, gender and SES. Cohen’s Kappa [28] was used to compare the agreement between the LR and MR models. The efficacy of each selection model was compared by the selection threshold of ATAR and UMAT, i.e. the lowest ATAR mark and UMAT score required to be selected for interview. There is overwhelming evidence for a positive correlation between UMAT score and ATAR marks and the risk for failure in medical programmes increases as these marks and scores decrease [2, 9, 10].

## Results

Data from 7735 applicants (53% females) who were eligible for interview (UMAT ≥50 and ATAR ≥96) were included in this study (Table 2). Overall, the number of eligible applicants varied across the years. Average SES level and UMAT scores were similar across cohorts. ATAR marks increased slightly across the cohorts ([Table 3]).

**Table 2:** Number of eligible applicants by cohort

Year	Male	%	Female	%	Total
2013	691	45%	835	55%	1526
2014	753	48%	832	52%	1585
2015	505	50%	501	50%	1006
2016	674	45%	820	55%	1494
2017	553	47%	623	53%	1176
2018	468	49%	480	51%	948
Total	3644	47%	4091	53%	7735

**Table 3:** Comparison of cohorts by UMAT, ATAR and SES

Measure	Statistics		2013	2014	2015	2016	2017	2018
UMAT	Mean		59.86	58.88	59.10	59.18	58.76	59.24
	95% CI	Lo	59.55	58.62	58.79	58.90	58.47	58.92
		Hi	60.17	59.14	59.41	59.45	59.05	59.57
	Median		59.00	58.67	59.00	59.00	58.33	59.00
	Variance		35.50	26.05	24.40	27.25	25.10	25.97
	Std. Deviation		5.96	5.10	4.94	5.22	5.01	5.10
	Minimum		50.00	50.00	50.00	50.00	50.00	50.00
	Maximum		89.00	79.67	78.67	80.67	76.33	83.33
	Skewness		0.71	0.50	0.53	0.52	0.57	0.50
	Kurtosis		0.48	0.07	0.35	0.32	0.29	0.31
ATAR	Mean		97.84	97.90	98.00	98.06	98.20	98.46
	95% CI	Lo	97.73	97.79	97.87	97.95	98.09	98.35
		Hi	97.95	98.00	98.12	98.17	98.31	98.57
	Median		98.61	98.70	98.75	98.85	98.90	99.10
	Variance		4.48	4.57	4.17	4.33	3.66	3.06
	Std. Deviation		2.12	2.14	2.04	2.08	1.91	1.75
	Minimum		91.05	91.00	91.00	91.00	91.05	91.10
	Maximum		99.95	99.95	99.95	99.95	99.95	99.95
	Skewness		−1.25	−1.29	−1.37	−1.39	−1.50	−1.87
	Kurtosis		0.80	0.82	1.23	1.23	1.70	3.43
SES	Mean		7.75	7.64	7.71	7.49	7.86	7.91
	95% CI	Lo	7.62	7.50	7.55	7.34	7.71	7.76
		Hi	7.89	7.77	7.87	7.63	8.00	8.07
	Median		9.00	9.00	9.00	9.00	9.00	9.00
	Variance		6.92	7.06	6.79	7.78	6.50	5.87
	Std. Deviation		2.63	2.66	2.61	2.79	2.55	2.42
	Minimum		1.00	1.00	1.00	1.00	1.00	1.00
	Maximum		10.00	10.00	10.00	10.00	10.00	10.00
	Skewness		−1.20	−1.12	−1.19	−1.01	−1.28	−1.34
	Kurtosis		0.38	0.21	0.45	−0.13	0.70	1.02

**Fig. 1:**
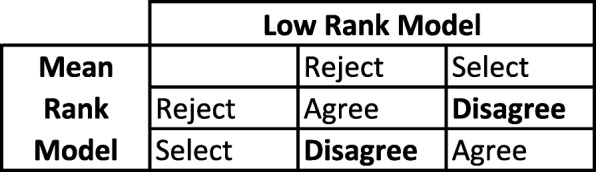
Selection groups

After applying the two models (for each cohort separately) to select applicants for interview (CM & NCM), four discrete groups were identified (Fig. 1). Of particular interest were the two groups that were selected by one model and not by the other. The results are presented for the six cohorts combined, although obviously, the selection algorithms were applied to each cohort separately.

The selection algorithms were set up to select the top 400 applicants for an interview in each year although in practice, the number of applicants selected for interview for UNSW Medicine ranged between 350 to 450 across those years. The outcomes of the selection algorithm demonstrate that overall there was about 9.4% disagreement between the two models, yielding Kappa >.79 across all cohorts (Table 4). The main interest therefore, lies within the difference between the applicants that were selected by one model and not by the other (Table 5).

**Table 4:** Selection outcomes by model by year

			Compensatory model (CM)					
	Year		Reject	Select	Reject	Select	N	Kappa*
**Nom-compensatory model (NCM)**	2013	Reject	1067	60	69.9%	3.9%	1526	0.796
		Select	60	339	3.9%	22.2%		
	2014	Reject	1132	53^#^	71.4%	3.3%	1585	0.821
		Select	54^#^	346	3.4%	21.8%		
	2015	Reject	556	51^#^	55.3%	5.1%	1006	0.790
		Select	50^#^	349	5.0%	34.7%		
	2016	Reject	1049	46^#^	70.2%	3.1%	1494	0.841
		Select	47^#^	352	3.1%	23.6%		
	2017	Reject	723	54	61.5%	4.6%	1176	0.795
		Select	54	345	4.6%	29.3%		
	2018	Reject	507	42	53.5%	4.4%	948	0.818
		Select	42	357	4.4%	37.7%		
	Total	Reject	5034	306^#^	65.1%	4.0%	7735	0.815
		Select	307^#^	2088	4.0%	27.0%		

*p < .00001 for all cohorts

^#^Inconsistency is due to having two or more applicants with the same final rank

**Table 5:** Group characteristics by model decision outcome

	CM	NCM	Min	Max	Mean	N	STD	95%CI of the Mean Lo Hi	
**UMAT**	Select	Reject	53.67	76.33	62.27	306^b^	5.28	61.68	62.86
	Reject	Select	57.67	63.33	59.93	307^b^	1.26	59.79	60.07
	Reject	Reject	50	79	56.82	5034	4.15	56.71	56.94
	Select	Select	57.67	89	64.19	2088	4.12	64.02	64.37
**ATAR**	Select	Reject	95.9	99.95	99.16	306^b^	0.84	99.07	99.26
	Reject	Select	98.2	99.7	99.11	307^b^	0.29	99.08	99.14
	Reject	Reject	91	99.95	97.27	5034	2.13	97.21	97.33
	Select	Select	98.2	99.95	99.61	2088	0.31	99.6	99.63
**SES**^a^	Select	Reject	1	10	7.97	296^b^	2.38	7.7	8.24
	Reject	Select	1	10	7.74	297^b^	2.77	7.42	8.05
	Reject	Reject	1	10	7.6	4816	2.69	7.52	7.68
	Select	Select	1	10	7.93	2013	2.49	7.82	8.04

^a^SES data was not available for all applicants

^b^Inconsistency is due to having two or more applicants with the same final rank

Table 5 demonstrates that the NCM yielded a higher threshold for selection compared to the CM. Across the entire six cohorts, the threshold for selection for interview set by the CM was UMAT ≥53.67 and ATAR ≥95.9, whereas applying the NCM model set the threshold much higher (UMAT ≥57.67 and ATAR ≥98.2). On the other hand, there was no difference in the minimum SES level across the same groups (SES ≥1) and the mean SES for the NCM was slightly lower compared to the CM (7.74 vs. 7.97,n.s.) demonstrating that raising the threshold did not disadvantage applicants from low SES backgrounds (Table 5).

## Discussion

This study addressed a previously overlooked topic in research into medical student selection, i.e. the efficacy of approaches to short-listing for selection interviews. Many applicants have the aptitudes to become good medical students and doctors, but fail to secure a place in the program [13, 29, 30]. In this study, two models of selection for interview were compared. The first is a compensatory model, which ranks applicants by their mean rank order of an aptitude test (UMAT) and secondary school achievement (ATAR); and the second is a non-compensatory model, ranking applicants by their lowest rank in either ATAR or UMAT. If two or more applicants have the same lowest rank, they are then ranked by their highest rank in either measure. In both models, UMAT scores and ATAR marks were modified to ranks, thus both measures had the same ‘weight’ and both were placed on the same scale. The comparison between the models was made only between applicants who were selected by one model and not by the other, as those who were selected by both or rejected by both were identical.

The main finding was that to be offered an interview via the NCM, higher UMAT scores and ATAR marks were required, compared with the compensatory model (CM). Interestingly however, the mean UMAT scores of the ‘CM select and NCM reject’ category were significantly higher than that of the ‘CM reject and NCM select’ group (Table 5). There was no meaningful difference in the mean ATAR marks between students selected for interview by each model (Table 5).

It has already been shown that to be successful in the medicine program, students need high marks/scores on both academic achievement and aptitude tests [7]. Consequently, the main risk factor for failure or struggling in medicine programs is associated with low UMAT scores and ATAR marks [2, 4, 11],it is suggested that the non-compensatory model is preferable from an academic perspective. The pool of applicants invited for interview would include only a few who would not meet the estimated threshold for success [2, 7] Tables 3.

Should the CM be used, the pool of applicants invited for an interview whose ATAR and UMAT results are below the threshold would be much larger. This finding suggests that the NCM selects applicants who have higher achievements on both UMAT and ATAR, and may reject applicants who have very high achievement in either the UMAT or the ATAR, but low achievement on the other measure. Nonetheless, medical schools also bear significant social accountability and wish to enhance diversity by admitting students from socioeconomically disadvantaged backgrounds. Moreover, it has been reported that ATAR and UMAT results are positively correlated with socioeconomic status (SES) [20, 31, 32]. Thus, it is important to identify the impact of the NCM and CM on the SES of applicants selected for interview. The results (Table 5) demonstrate that there was no statistically significant difference in SES between NCM and CM, yet the NCM yielded selected applicants that on average had slightly lower SES. This result is important since it demonstrates that it is possible to raise the academic threshold for selection to medicine without having any negative impact on applicants from low SES backgrounds.

Overall, the evidence gathered in this study suggests that a non-compensatory model is preferable for selecting applicants for medical student selection interview.

This study has a number of potential limitations. The first limitation is the generalisability of the results. Would equivalent results be expected if a similar study was applied to a different population? A comparison of the thresholds yielded by the NCM over six cohorts of applicants (Table 5) with thresholds identified in a large multi-national, multi cohort study [2] strongly suggest that a non-compensatory model would be effective, at least in Australia and New Zealand, and most likely globally. However, it is recommended that similar analyses should be performed employing data from other jurisdictions to assess the generalisability of this study.

Some may also question the methodology of the analyses, which focused on thresholds rather than means. This study focused on the thresholds for selection, since these are the most critical measures that determine whether an applicant is selected for interview or not; and within the pre-medicine program achievement range where the risk for failure is greatest [1, 2, 9]. Conversely, the means of scores focus on the central representative measure of the applicants, which is well above the threshold for selection. Therefore, any difference in the means may not necessarily impact applicants with achievements around the threshold range.

## Conclusion

This study demonstrated that it is possible to raise the threshold for medical student selection tools without having negative impact on socioeconomically disadvantaged applicants. This can be achieved by applying a non-compensatory method to determine selection for interview. The scarcity of studies investigating the validity of the selection process for interview suggest that there is a need for more research into this topic, particularly since any improvement in the suitability and quality of the pool of applicants being interviewed would very likely improve the quality of selected medical students.

## Data Availability

Anonymised data are stored on UNSW secure server. Data are available by request conditional to the approval of UNSW ethics committee.

## Abbreviations

UMAT: Undergraduate Medicine and Health Sciences Admission Test
ATAR: Australian Tertiary Admission Rank
CM: Compensatory model
NCM: Non-compensatory model
